# Research progress on alternative non-classical mechanisms of PCSK9 in atherosclerosis in patients with and without diabetes

**DOI:** 10.1186/s12933-020-01009-4

**Published:** 2020-03-13

**Authors:** Ying Tang, Sheng-Lan Li, Jia-Hui Hu, Kai-Jun Sun, Lei-Ling Liu, Dan-Yan Xu

**Affiliations:** Department of Internal Cardiovascular Medicine, The Second Xiangya Hospital, Central South University, 139 Middle Renmin Road, Changsha, 410011 Hunan China

**Keywords:** PCSK9, Atherosclerosis, LDL-C, Inflammation, Mitochondrial DNA

## Abstract

The proprotein convertase subtilisin/kexin type 9 (PCSK9) acts via a canonical pathway to regulate circulating low-density lipoprotein-cholesterol (LDL-C) via degradation of the LDL receptor (LDLR) on the liver cell surface. Published research has shown that PCSK9 is involved in atherosclerosis via a variety of non-classical mechanisms that involve lysosomal, inflammatory, apoptotic, mitochondrial, and immune pathways. In this review paper, we summarized these additional mechanisms and described how anti-PCSK9 therapy exerts effects through these mechanisms. These additional pathways further illustrate the regulatory role of PCSK9 in atherosclerosis and offer an in-depth interpretation of how the PCSK9 inhibitor exerts effects on the treatment of atherosclerosis.

## Background

High plasma levels of low-density lipoprotein-cholesterol (LDL-C) is a predominant risk factor for atherosclerosis. The proprotein convertase subtilisin/kexin type 9 (PCSK9) has been reported to play an important role in the development of atherosclerosis; PCSK9 monoclonal antibodies (mAbs), including evolocumab and alirocumab, have been put into clinical use to decrease circulating PCSK9 [1, 2]. In addition, a small interfering RNA (siRNA) molecule, inclisiran, decreases the hepatic production of PCSK9 [3]. PCSK9 secreted by the liver combines with the epidermal growth factor-like repeat A (EGF-A) domain of the LDL receptor (LDLR) to form the PCSK9–LDLR complex, which is internalized by endosomes and then undergoes a degradation process in lysosomes. Thus, the reduced amount of LDLRs on the surface of liver cells causes a decrease in the clearance of circulating LDL-C.

Apart from this classical pathway, recent studies have described other roles played by PCSK9 in atherosclerosis. In the present review, we will focus on these alternative mechanisms in detail. Specifically, we will first describe the role of extrahepatic PCSK9 expression and other PCSK9-mediated pathways involved in lipid metabolism. Then we discuss the role of PCSK9 in macrophage cholesterol efflux, apoptosis of endothelial cells, mitochondrial dysfunction, and inflammatory mechanisms of atherogenesis. Finally, we will highlight clinical treatment with PCSK9 mAbs and offer directions for future research.

## The role of extrahepatic PCSK9 expression in promoting atherosclerosis

The expression of PCSK9 is initially regulated at the transcriptional level by sterol regulatory element-binding proteins (SREBPs), which regulates genes involved in cholesterol metabolism (SREBP-2) and fatty acid synthesis (SREBP-1c) [4]. Resveratrol is a polyphenolic compound that protects against atherosclerosis [5]. Jing et al. [6] found that hepatic PCSK9 expression could be attenuated by resveratrol via downregulating the expression of SREBP-1c. In addition, hepatic PCSK9 expression is upregulated in conditions of excessive dietary fat consumption [7]. This indicates that hepatic PCSK9 expression can be modulated by nutritional status; this regulatory process is also a SREBP-1c-mediated pathway [8].

Although predominantly expressed in the liver, PCSK9 is also expressed in extrahepatic tissues such as the intestine, kidneys, and blood vessels. PCSK9 secreted by the kidney and blood vessels enters circulation and downregulates the LDLR levels of other cells, including hepatocytes and macrophages, thus lowering plasma LDL-C uptake of these cells [9, 10]. In contrast, in the intestine, PCSK9 upregulates the cholesterol level mainly by reducing the secretion rather than the uptake of plasma LDL-C [11, 12]. Based on these mechanisms, LDL-C primarily accumulates in the plasma and results in the development of atherosclerosis.

May et al. [11] reported that PCSK9 was expressed in the full length of the small intestine and colon, with the expression values being almost the same along the intestinal cephalo-caudal axis. Further, administration of PCSK9 obviously decreased the LDLR content of the duodenum and transintestinal cholesterol excretion in PCSK9 knockout (PCSK9^−/−^) mice [12]. Administration of ^3^H-LDL increased the plasma ^3^H-cholesterol level both in PCSK9^−/−^ and wild-type (WT) mice to a similar extent, but the subsequent cholesterol content was lower in the PCSK9^−/−^ mice because of faster LDL-C clearance [12]. These findings indicate that PCSK9 increases the plasma cholesterol content by reducing transintestinal cholesterol excretion. In addition, TG-rich lipoprotein (TRL) remnants are known to directly mediate the pathogenesis of atherosclerosis. Research has shown that knockout of PCSK9 in mice clearly inhibited postprandial hypertriglyceridemia [11]. Patients with PCSK9 loss-of-function mutations displayed lower postprandial TG levels [13], indicating that PCSK9 may induce an increase in the plasma TG levels by stimulating the production of TRL in the intestine. Moreover, clinical research has found that the PCSK9 mAbs, including evolocumab and alirocumab, showed no influence on the postprandial lipemia peak in normolipidemic individuals [1, 2]. However, in individuals with hypertriglyceridemia, evolocumab played a modest role in lowering TG levels [14, 15], indicating that anti-PCSK9 mAbs may impact TG levels in a dose-dependent manner.

Luo et al. [9] found that expression of the human PCSK9 transgene in the murine kidney resulted in an elevation in the plasma PCSK9 levels to ten times the levels in human plasma. The authors also confirmed that nearly no hepatic LDLR remained and the circulating LDL-C levels were markedly increased in the transgenic mice. Taken together, these findings illustrate that PCSK9 expression in the kidney and its entry into the plasma resulted in considerably accelerated LDLR degradation in the liver, leading to an increase in the plasma LDL-C level.

In 2012, Ferri et al. [10] found that PCSK9 was present in blood vessel cells and human atherosclerotic plaques. Further, it was demonstrated that smooth muscle cells (SMCs) were the only cells in human vascular tissues that expressed and secreted PCSK9. Due to the deficiency of LDLR expression in SMCs, Ferri et al. used a co-culture of SMCs and macrophages to examine the effects of PCSK9 secreted by SMCs; they found that the LDLR content on macrophages decreased, but LDLR expression in the macrophages was dramatically increased when the SMCs were transfected with PCSK9-siRNA. These results demonstrate that PCSK9 secreted from human SMCs caused a decrease in LDLR expression of macrophages; therefore, PCSK9 secreted by blood vessel cells may also play a role in the formation of atherosclerotic plaques.

PCSK9 exists both intracellularly and extracellularly. Circulating PCSK9 is secreted from hepatocytes and other cells. PCSK9 mAbs mainly decreases circulating PCSK9, while inclisiran targets hepatic production of PCSK9 [3]. Accordingly, the mechanisms of PCSK9 in atherosclerosis can be grouped into intracellular and extracellular mechanisms (Table 1).

**Table 1:** Intracellular and extracellular mechanisms mediated by PCSK9

PCSK9 location	Therapeutic agents targeting PCSK9	Mechanisms mediated by PCSK9
Intracellular	siRNA (inclisiran)	1. LDLR degradation 2. Inhibiting the degradation of apoB100 3. Inducing mitochondrial dysfunction 4. Inducing endothelial cell apoptosis
Extracellular (circulating/plasma)	PCSK9 mAbs (evolocumab, alirocumab)	1. LDLR degradation 2. VLDLR degradation 3. Positively correlated with chronic inflammation 4. Positively associated with platelet count/fibrinogen levels

## Other PCSK9-mediated pathways involved in lipid metabolism

Apart from the canonical pathway, PCSK9 also regulates lipid metabolism through the following mechanisms: intracellular endogenous PCSK9-induced LDLR degradation, VLDLR degradation, regulation of apoB secretion, and Lp(a) metabolism.

### Intracellular endogenous PCSK9-induced LDLR degradation

**Fig. 1:**
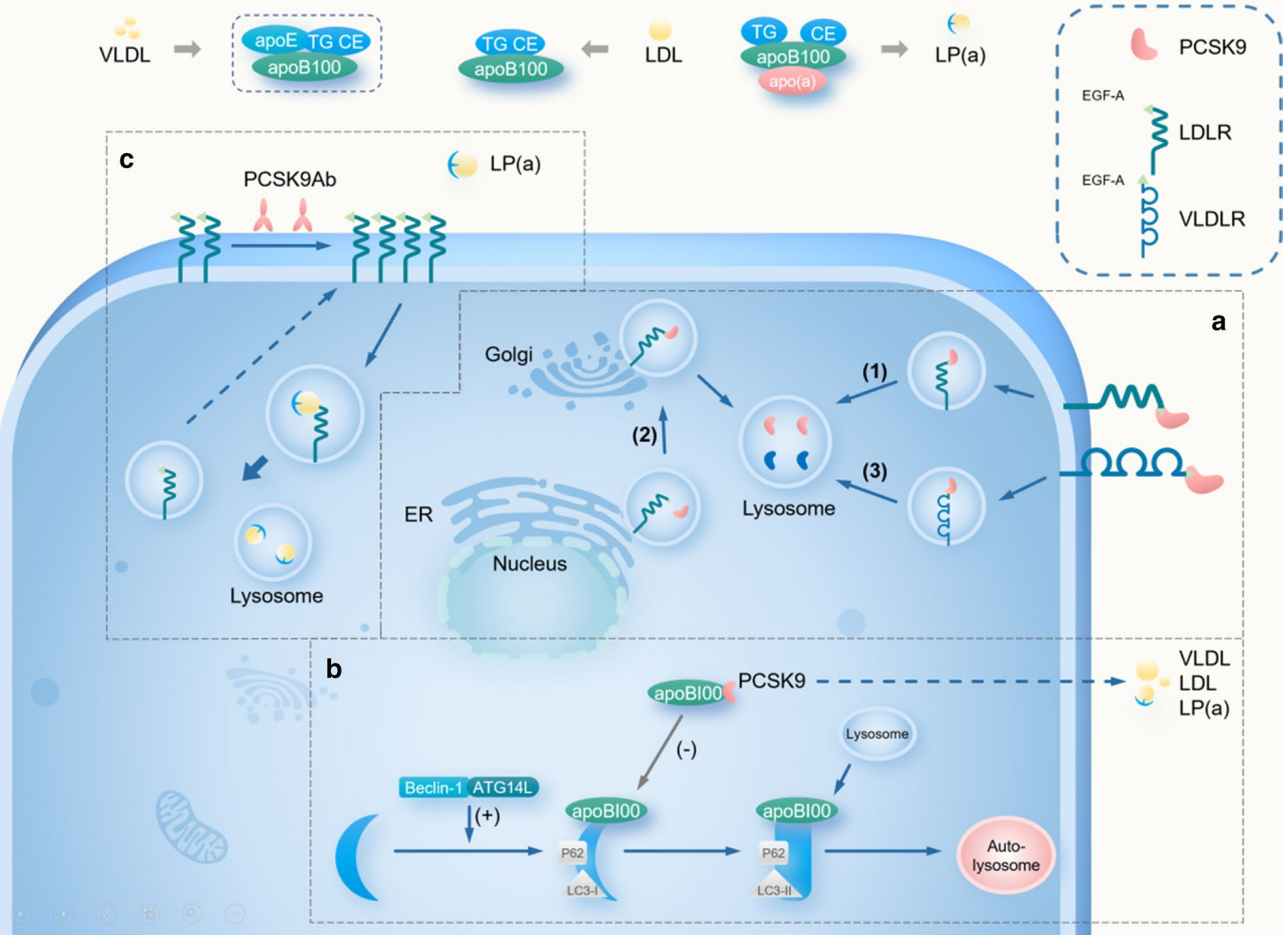
PCSK9 in lipid metabolism. **a** (1) The canonical pathway: secreted PCSK9 can bind to the EGF-A domain of low-density lipoprotein receptor (LDLR) on the cell surface and be internalized with LDLRs in endocytic vesicles. The PCSK9–LDLR complex is then degraded in lysosomes. (2) Intracellular endogenous PCSK9-induced LDLR degradation: PCSK9 can bind to LDLR in the luminal secretory compartment and target LDLR to lysosomes in vesicles emanating from the trans-Golgi. (3) PCSK9-mediated very low-density lipoprotein receptor (VLDLR) degradation: the EGF-A domain of VLDLR is homologous to the EGF-A domain of LDLR. Extracellular PCSK9 interacts with VLDLR by binding with the EGF-A domain to form an endocytosed complex that is degraded in lysosomes. **b** The Beclin-1/ATG14L complex initiates the autophagy process that degrades apolipoprotein B (apoB). P62 shuttles proteins and organelles to autophagosomes for degradation. The conversion of LC3-I to LC3-II indicates active turnover of the autophagy process. PCSK9 interacts with apoB, resulting in the inhibition of the degradation pathway of apoB via this autophagic mechanism. Thus, the secretion of apoB100, as the particles VLDL, LDL, and Lipoprotein(a) (Lp(a)), into circulation is increased. PCSK9 antibodies increase the expression of LDLR, which can bind with exogenous Lp(a). Thus, the Lp(a)/LDLR complex is internalized and undergoes the lysosome degradation pathway

Autosomal recessive hypercholesterolemia (ARH) is due to the lack of the functional ARH protein. ARH protein is essential for the internalization of the LDLR by binding with the intracellular domain of the LDLR. However, even in the absence of the ARH protein, PCSK9 still resulted in a decrease in the level of LDLR [16]. Treatment with evolocumab further decreased the LDL-C levels in ARH patients with very low levels of cell surface LDLR internalization [17, 18]. These findings are indicative of the existence of an ARH-independent intracellular approach for the PCSK9-mediated degradation of LDLR. Further, Poirier et al. [19] observed that the LDLR levels rapidly increased in HepG2 cells when cytoplasmic transport from the trans-Golgi to lysosomes was blocked by siRNA acting against the clathrin light chain. However, the LDLR levels were not significantly affected when blocking the canonical lysosomal degradation pathways [19], suggesting that endogenous PCSK9 can directly bind with LDLR in the Golgi network to induce the lysosomal degradation of LDLR. Thus, endogenous PCSK9 can induce LDLR degradation via an intracellular pathway (Fig. 1a).

### PCSK9-mediated VLDLR degradation

PCSK9 is also involved in the metabolic activity of very low-density lipoprotein receptor (VLDLR). Poirier et al. [20] found that culturing VLDLR-expressing cells in a PCSK9-containing medium resulted in a decrease in the VLDLR level. Roubtsova et al. [21] also observed that the VLDLR level was improved by 40-fold in PCSK9^−/−^ mice compared with WT mice; this further confirms that PCSK9 is involved in the degradation of VLDLR. Furthermore, in LDLR and PCSK9 knockout (LDLR^−/−^PCSK9^−/−^) mice, a similar 36-fold increase in the VLDLR level was observed [21]. These findings indicate that PCSK9 regulated the VLDLR levels in an LDLR-independent manner. With regard to the underlying mechanism, studies on the structure of VLDLR have identified an EGF-A domain that is homologous to the EGF-A domain in LDLR. Moreover, the binding between PCSK9 and VLDLR could be eased by receptor-specific antibodies or by a synthetic LDLR EGF-A peptide [22]. Thus, the mechanism of PCSK9 in the regulation of VLDLR seems to be similar to that observed in LDLR degradation, specifically, PCSK9 interacts with VLDLR by binding with the EGF-A domain to form an endocytosed complex that is degraded via lysosomal pathways (Fig. 1a).

### PCSK9-mediated stimulation of apolipoprotein B secretion

Apolipoprotein B (apoB) can carry LDL. Abnormally high levels of apoB lead to pathological inflammation, which is a major factor associated with atherogenesis. Research has found that PCSK9 with gain-of-function mutations induces an increase in the level of LDL, which is associated with the secretion of apoB100 [23]. The secretion of both apoB100 and apoB48 was increased in human enterocytes that were treated with recombinant human PCSK9; this indicates that PCSK9 had a stimulatory effect on apoB secretion [24]. Similarly, Tavori et al. [25] compared the plasma apoB levels in transgenic mice carrying human PCSK9 (hPCSK9tg mice) and WT mice, and found that hPCSK9 resulted in an increase in hepatic apoB secretion. In addition, they reported a higher apoB level in hPCSK9tg/LDLR^−/−^ mice than in WT/LDLR^−/−^ mice, indicating that the overexpression of apoB was related to PCSK9 regardless of LDLR expression [25]. Likewise, Sun et al. [26] demonstrated that both PCSK9-overexpressing WT and LDLR^−/−^ mice exhibited increased production of apoB. These results indicate that PCSK9 induces increased apoB secretion through an LDLR-independent pathway, thereby influencing the process of atherogenesis.

Autophagy is known to be a dynamic regulatory factor in apoB degradation [27]. Beclin-1 and ATG14L form a complex to initiate the autophagic process; p62 transfers organelles and proteins to the autophagosomes and the transformation of LC3-I to LC3-II occurs in an active autophagy process. Sun et al. [26, 28] found that the deletion of PCSK9 in atherosclerosis-prone Apobec1^−/−^LDLR^−/−^ mice caused the reduction in hepatic apoB secretion and the inhibition of the development of atherosclerosis. In Apobec1^−/−^LDLR^−/−^PCSK9^−/−^ mice, the accumulation of Beclin-1 and p62 was markedly lower due to greater consumption of Beclin-1 and p62 and the conversion of LC3-I to LC3-II was increased over fourfold [26, 28]. Collectively, these results indicate the presence of a more active autophagy process in the absence of PCSK9. Thus, PCSK9 may promote atherogenesis by negatively modulating the autophagy-signaling pathway and autophagic degradation activity (autophagic flux) to increase the secretion of apoB (Fig. 1b).

### PCSK9-mediated lipoprotein(a) metabolism

Lipoprotein(a) (Lp(a)) is an atherogenic LDL-like particle identified by the presence of apoprotein(a) (apo(a)), which is covalently bound to apoB100 on an LDL particle [29]. A longitudinal observational study that included 516 patients with at least two cardiovascular risk factors reported that a higher Lp(a) level was an independent risk factor for atherosclerotic cardiovascular disease [30]. Lp(a) levels were positively correlated with the amount of narrow coronary vessels, indicating that the Lp(a) level is proportional to the severity of coronary atherosclerosis [31]. Moreover, Tavori et al. [32] found that PCSK9 could bind with Lp(a) to form an Lp(a)-PCSK9 complex in individuals with high Lp(a) contents. Carriers of R46L, a PCSK9 loss-of-function mutation, have a lower level of Lp(a) and reduced cardiovascular risk compared with noncarriers [33, 34]. Numerous studies have found that the inhibition of PCSK9 with monoclonal antibodies decreased the content of LDL-C and the plasma Lp(a) level [35–38]. When the Lp(a) level is high, PCSK9 preferentially binds with Lp(a) rather than LDL [32]; however, the detailed mechanisms by which PCSK9 and its inhibitors participate in the metabolism of Lp(a) require further research.

PCSK9 augments the secretion of apo(a) and apoB100 [24, 26], which are the prerequisites for Lp(a) assembly, so the inhibition of PCSK9 could dampen these effects, thus reducing the production of Lp(a) [39, 40]. Treatment with alirocumab resulted in an 18.7% reduction of Lp(a), and this reduction was parallel to a 24.6% increase of fractional clearance rates for apo(a), suggesting that PCSK9 inhibitors increase the clearance of Lp(a) through apo(a) degradation [1]. However, Tavori et al. [32] reported that immunoprecipitation of PCSK9 from the plasma of transgenic mice expressing recombinant apo(a) failed to pull down apo(a), indicating that PCSK9 does not diametrically bind with apo(a) to exert effects on Lp(a) metabolism. Another study found that exogenous PCSK9 induced a robust decrease in the internalization of apo(a) and Lp(a), whereas overexpression of LDLR increased the internalization of Lp(a) dramatically, indicating that PCSK9 may interfere with Lp(a) internalization via degrading LDLR [41]. Treatment with the lysosomal inhibitor in HepG2 cells caused the cytoplasmic accumulation of apo(a)/Lp(a) [41]. Thus, it can be inferred that PCSK9 dampens apo(a)/Lp(a) internalization by degrading LDLR, and the internalized Lp(a)/apo(a) goes through the lysosome degradation pathway (Fig. 1c).

If the inhibition of PCSK9 indeed lowers Lp(a) only in the LDLR-dependent clearance pathway, the change in Lp(a) would likely be proportional to the change in LDL-C. However, a clinical trial reported that treatment with evolocumab caused a discordance between Lp(a) and LDL-C reductions, suggesting that other mechanisms mediated by PCSK9 might also participate in Lp(a) catabolism [42]. Further, statins, which could increase LDLR expression, were reported to have little effect on lowering Lp(a) [43], and may even cause a significant increase in the Lp(a) level after statin therapy [44]. These findings reveal that LDLR levels may only be minimally related to PCSK9-mediated Lp(a) clearance. Generally, the plasma Lp(a) concentration is negatively correlated with apo(a) isoform size [45], and individuals with lower baseline Lp(a) concentration showed greater Lp(a) reduction after evolocumab prescription, indicating that larger apo(a) isoforms may play a greater role in PCSK9-induced Lp(a) clearance [42].

## The role of PCSK9 in macrophage cholesterol efflux

Cholesterol efflux in macrophages, i.e., reverse cholesterol transport (RCT), is one of the most important mechanisms preventing atherosclerosis. RCT is primarily induced by ATP-binding cassette transporter A1 (ABCA1), which operates as a harvester of cholesterol in the cell and delivers it from the endosome and lysosome to the cell membrane, where it binds with apolipoprotein A-I. PCSK9 can accelerate atherosclerosis by reducing ABCA1 expression in the macrophages, thereby inhibiting RCT [46, 47].

Denis et al. [46] reported the attenuation of atherosclerotic phenotypes as well as four-fold lower aortic cholesteryl ester accumulation due to the more active RCT process in PCSK9^−/−^ mice than in WT mice. Therefore, a potential mechanism by which PCSK9 regulates atherosclerosis could target RCT. Further, Adorni et al. [47] found that the overexpression of PCSK9 inhibited the expression of ABCA1; therefore, ABCA1-mediated cholesterol efflux was also inhibited. Interestingly, PCSK9 failed to inhibit cholesterol efflux in LDLR^−/−^ mice [47]. These findings indicate that PCSK9 downregulates ABCA1 expression to inhibit ABCA1-mediated RCT dependent of LDLR.

## The role of PCSK9 in endothelial cell apoptosis

Endothelial damage serves as a trigger of atherosclerosis. Excessive endothelial cell apoptosis alters endothelial integrity and increases permeability, thus facilitating endothelial dysfunction and the occurrence of atherosclerosis. PCSK9 induces endothelial cell apoptosis through the Bcl-2/Bax–Caspase9–Caspase3 mitochondrial pathway and the p38/JNK/MAPK signaling pathway to alter the integrity of the endothelium, ultimately promoting endothelial dysfunction and the development of atherosclerosis.

Wu et al. [48] investigated the mechanism by which PCSK9 modulates the apoptosis of human umbilical vein endothelial cells (HUVECs). Specifically, HUVECs were incubated with oxidized-LDL (ox-LDL) for 1 day after transfection with PCSK9 siRNA, and the authors detected the expression of Bcl-2, Bax, Caspase3, and Caspase9 [48]. Bax is a pro-apoptotic protein that could increase the permeability of the outer membrane of mitochondria by forming pores in the membrane; Bcl-2 exerts the opposite effect, thus it is an anti-apoptotic protein. The ratio of Bcl-2 to Bax indicates the ability of cells to undergo apoptosis. The study found that HUVECs that were pretreated with PCSK9 siRNA exhibited considerably reduced cell apoptosis and had a decreased expression of the pro-apoptotic proteins Bax, Caspase3, and Caspase9 [48]. At the same time, the level of the anti-apoptotic protein Bcl-2 was increased. According to these findings, it was speculated that PCSK9 mediated ox-LDL-induced apoptosis via the Bcl-2/Bax–Caspase9–Caspase3 mitochondrial pathway, thus reducing the ratio of Bcl-2/Bax, increasing Caspase3 and Caspase9 activities, and ultimately increasing the sensitivity of endothelial cells to apoptosis.

Another pathway by which PCSK9 may mediate apoptosis in atherosclerosis was found by Li et al. [49] in a study focused on whether the MAPK pathway participated in PCSK9-mediated endothelial cell apoptosis in atherosclerosis. They found that shRNA acting against PCSK9 significantly inhibited the phosphorylation of p38 and JNK [49], indicating that PCSK9 promoted the development and progression of atherosclerosis by mediating the apoptosis of endothelial cells via the p38/JNK/MAPK signaling pathway.

## The role of PCSK9 in inflammation

Li et al. [50] reported that plasma PCSK9 levels were positively associated with the white blood cell count in patients with coronary artery disease (CAD). In addition, the serum PCSK9 level was positively associated with the volume and fraction of necrotic core tissue of atherosclerotic plaques, which is responsible for coronary plaque inflammation [51]. Thus, PCSK9 is positively correlated with chronic inflammation, which plays an important role in inducing atherosclerosis. In fact, research [52] has confirmed the interaction between PCSK9 and inflammation, namely, that inflammation has been found to stimulate the expression of PCSK9. For example, Feingold et al. [52] found that LPS administration resulted in an increase in the PCSK9 mRNA levels in cholesterol-fed mice, and this effect gradually increased with time. Thus, inflammation was directly associated with increased PCSK9 expression. Likewise, other compounds associated with inflammation, such as turpentine and zymosan, have also been found to stimulate the expression of PCSK9 [52]. In turn, PCSK9 has been found to promote the development of inflammation, for which the following three main mechanisms have been reported:

### PCSK9-stimulated secretion of inflammatory cytokines

**Fig. 2:**
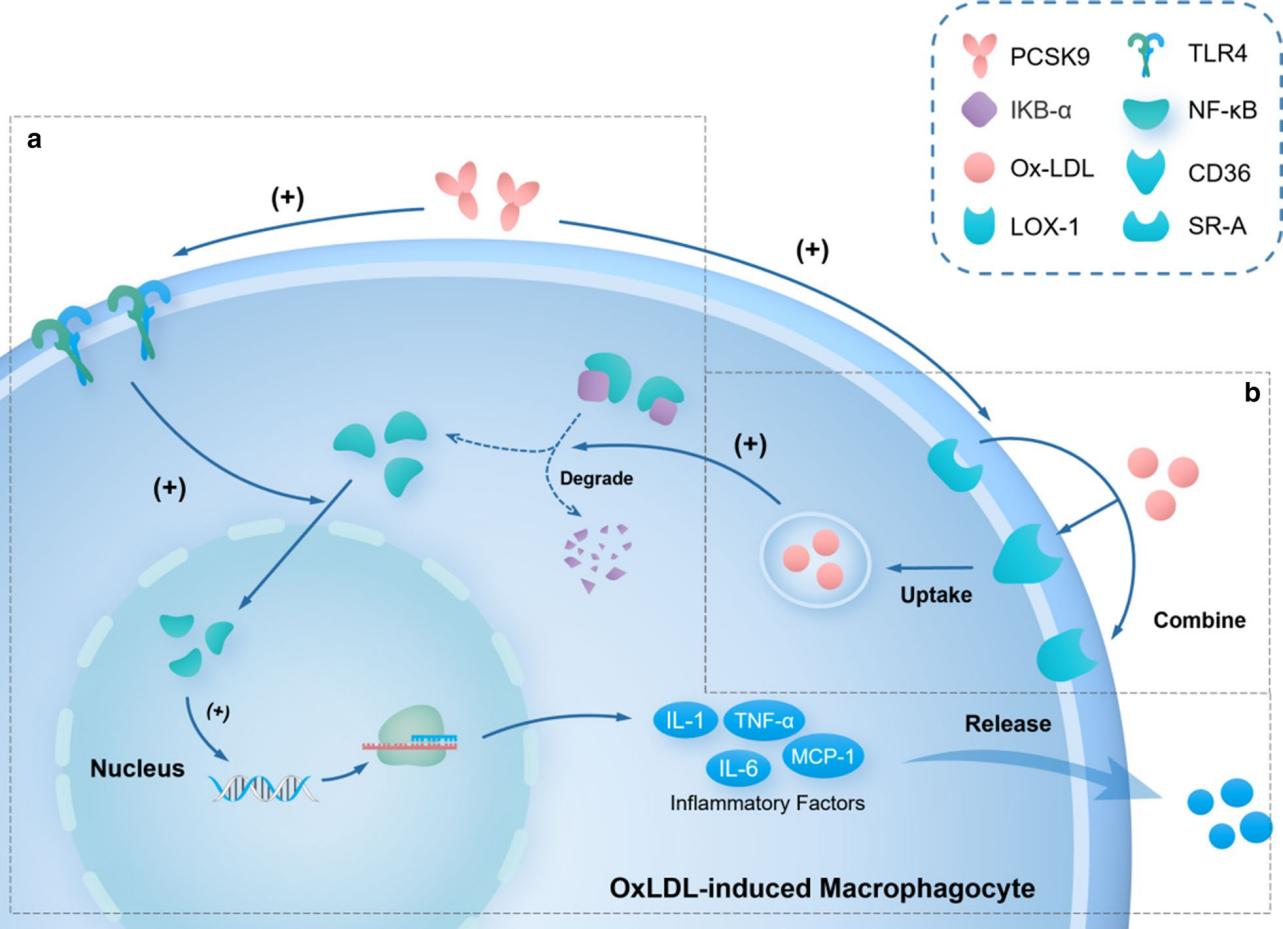
PCSK9-regulated expression of inflammatory cytokines and scavenger receptors. NF-κB is bound to IκBα in the cytoplasm in the resting state. With cell stimulation, such as with oxidized-LDL (ox-LDL), IκBα is degraded and the released NF-κB is then translocated into the nucleus to activate the transcription of target genes including inflammatory cytokines. In ox-LDL-induced macrophagocytes, PCSK9 enhances the expression of TLR4, which can promote NF-κB nuclear translocation. Then, the expression and secretion of inflammatory factors, including IL-1, IL-6, monocyte chemo-attractant protein 1 (MCP-1), and TNF-α, are increased. **a** PCSK9 promotes the expression of different scavenger receptors (SRs), including SR-A, CD36, and LOX-1 in macrophagocytes, thus enhancing ox-LDL uptake in macrophagocytes. **b** PCSK9 promotes expression of different SRs, including SR-A, CD36, and LOX-1 in macrophagocytes, thus enhancing ox-LDL uptake in macrophagocytes

OxLDL-induced inflammatory response of macrophages plays a key role in the pathogenesis of atherosclerosis [53]. Tang et al. [54] observed that PCSK9 siRNA suppressed ox-LDL-induced upregulation of IL-1α, IL-6, and TNF-α in a dose-dependent manner. In addition, PCSK9 siRNA reduced IκBα degradation and NF-κB nuclear translocation, which are responsible for the expression and secretion of inflammatory cytokines. These findings indicate that PCSK9 siRNA suppressed the release of inflammatory cytokines in ox-LDL-stimulated macrophages through inhibiting IκBα degradation and NF-κB nuclear translocation, thus providing protection against inflammation. Tang et al. [55] further investigated the pro-inflammatory effect of PCSK9. The authors found that the expression level of PCSK9 was increased in atherosclerotic plaques and that PCSK9-silenced mice exhibited fewer aortic atherosclerotic plaques, as well as decreased expression of vascular inflammation regulators, such as IL-1β, TNFα, monocyte chemo-attractant protein 1 (MCP-1), TLR4, and NF-κB, in the lesions [55]. Based on these findings, it appears that PCSK9 functions as an inflammatory mediator by exacerbating vascular inflammation via the TLR4/NF-κB signaling pathway in the pathogenesis of atherosclerosis (Fig. 2a).

### PCSK9-mediated regulation of the expression of scavenger receptors

A crucial component in the process of atherogenesis is scavenger receptors (SRs) on monocytes and macrophages that can bind with ox-LDL, a pro-inflammatory cytokine [56]. Ding et al. [57] reported increased levels of different SRs, including SR-A, CD36, and LOX-1, as well as ox-LDL uptake in macrophages treated with PCSK9. These findings illustrate that PCSK9 upregulates the expression of SRs to enhance ox-LDL uptake in monocytes and macrophages, thus promoting inflammation and the formation of atherosclerotic lesions (Fig. 2b).

### PCSK9-induced increase in the migratory capacity of monocytes

Monocytes, a type of inflammatory cell, play a role in atherosclerosis because of their ability to migrate to the arterial wall. Giunzioni et al. [58] found that PCSK9 progressively accumulated in atherosclerotic lesions, inducing an increased infiltration of inflammatory monocytes by 32% compared with the controls. These findings confirm that PCSK9 directly facilitated inflammation in atherosclerotic lesions via the recruitment of monocytes.

**Fig. 3:**
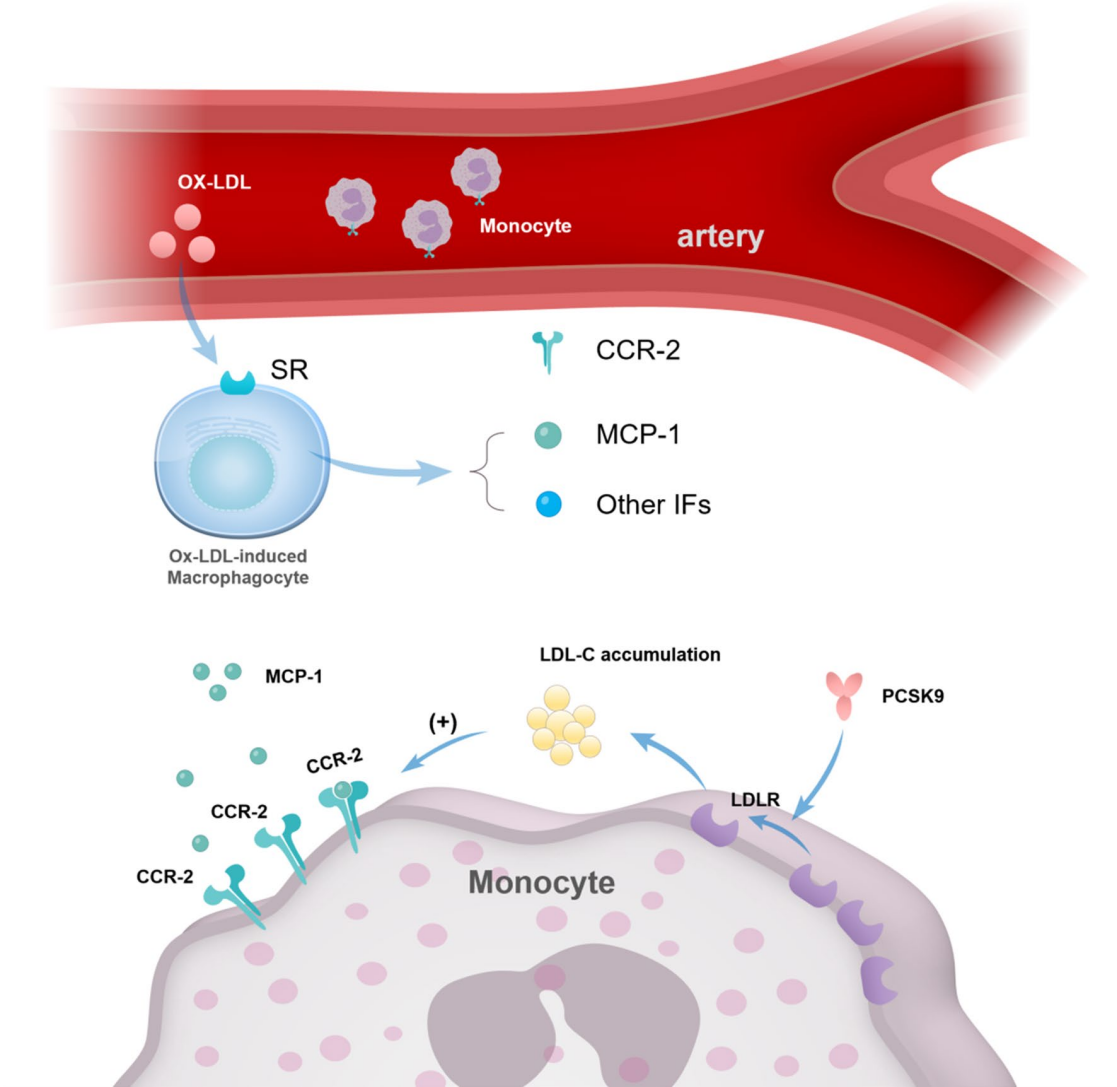
PCSK9-regulated migratory capacity of monocytes. PCSK9 decreases the LDLR on the cell surface of a monocyte. As a result, the accumulated circulating low-density lipoprotein-cholesterol (LDL-C) stimulates the expression of C–C chemokine receptor 2 (CCR2), which serves as the receptor of MCP-1. Thus, the monocyte can migrate towards MCP-1 and accumulate in the arterial wall

Studies have investigated the mechanism underlying the PCSK9-monocyte relationship in atherosclerosis. In one such study, the administration of PCSK9 mAbs efficiently masked the pro-inflammatory monocyte phenotype in patients with familial hypercholesterolemia; at the same time, the surface expression of the C–C chemokine receptor 2 (CCR2) and TNFα secretion were both downregulated. These findings indicate that PCSK9 mAbs inhibited inflammation by reducing the CCR-2-related migratory ability of monocytes [59]. In addition, Grune et al. [60] demonstrated that PCSK9 secreted from vascular smooth muscle cells (VSMCs) reduced LDLR expression on monocytes and subsequently increased LDL-C-augmented monocyte CCR2 expression. Based on these findings, the following hypothesis can be drawn: PCSK9 upregulates LDL-C-mediated CCR2 expression of monocytes by suppressing the level of LDLR; monocytes with high CCR2 expression migrate towards MCP-1 and accumulate in the arterial wall. As a result, the recruitment of multiple monocytes triggers arterial inflammation and mediates atherosclerosis (Fig. 3).

### Anti-PCSK9 therapy associated with high-sensitivity C-reactive protein (hs-CRP) levels

High-sensitivity C-reactive protein (hs-CRP) has been established as an inflammatory marker that can be used to predict the progression of atherosclerotic diseases [61]. Although multiple studies have identified pro-inflammatory effects of PCSK9 in promoting atherosclerosis, several studies have found that hs-CRP levels were not decreased despite the anti-inflammatory changes after treatment with PCSK9 inhibitors [62, 63]. This suggests that hs-CRP may be merely a predictor—not a mediator—for atherosclerotic diseases [64].

## The role of PCSK9 in mitochondrial dysfunction

Mitochondria are the main cellular components involved in the induction of oxidative stress. Previous studies have confirmed that excess mitochondrial-derived reactive oxygen species (mtROS) induced DNA, RNA, protein, and lipid peroxidation injury, which led to mitochondrial dysfunction and thereby facilitated the development of atherosclerosis [65]. Ding et al. [66] found that PCSK9 knockdown with siRNA inhibited mtROS production in VSMCs and endothelial cells by 50% and 30%, respectively. Based on this finding, it was proposed that mtROS generation followed the same pattern as PCSK9 expression, and their interaction was significant in the pathogenesis of atherosclerosis. In addition, Ding et al. [67] found that hPCSK9 increased mtROS generation and mtDNA damage in a near-linear dose-dependent manner. These observations indicate a bidirectional interplay between mtDNA damage and PCSK9 mediated by mtROS. Another study has shown that pretreatment of cells with mtROS inhibitors decreased the PCSK9 level and damage to the mtDNA. In addition, inhibition of DNase II, a lysosome that digests damaged mtDNA, enhanced both mtROS production and PCSK9 expression [67]. According to these findings, mtROS may be a link in the PCSK9-mtDNA interplay, through which PCSK9 expression, mtROS, and mtDNA damage form a positive feedback loop to synergistically facilitate cell injury, thus inducing atherosclerosis.

## Association of PCSK9 with platelet count/fibrinogen levels

Large amounts of procoagulant factors are released into vessel lumens after unstable atherosclerotic plaque rupture; this leads to the development of atherothrombosis and thereby triggers the onset of cardiovascular events. Platelets and fibrinogen, which are essential for thrombus formation, have a strong positive correlation with PCSK9.

Li et al. [68] explored the association between plasma platelet indices and PCSK9 levels. In their cross-sectional study on 330 stable CAD patients, the plasma levels of PCSK9 were positively associated with the platelet count; this indicates that the role of PCSK9 in atherosclerosis involves platelet-associated mechanisms. Moreover, fibrinogen is now acknowledged as a new biomarker of atherosclerotic diseases. Zhang et al. [69, 70] determined the plasma PCSK9 concentration and fibrinogen levels in 219 stable CAD patients. Their data demonstrated that patients with high PCSK9 levels also had high fibrinogen levels; thus, circulating PCSK9 and fibrinogen levels were positively correlated with atherosclerosis. However, the specific mechanisms underlying the interaction between PCSK9 and platelets/fibrinogen require further investigation.

## Association of PCSK9 mAbs with diabetes mellitus (DM)

PCSK9 mAbs significantly reduce the level of plasma LDL-C, effectively reducing the incidence of atherosclerosis. Alirocumab and evolocumab have been prescribed for those with uncontrolled LDL-C levels and familial hypercholesterolemia. In this section, we primarily focus on cardiovascular-protective and diabetogenic effects of PCSK9 mAbs in patients with and without diabetes mellitus (DM).

### PCSK9 mAbs reducing cardiovascular risk in patients with DM

Ray et al. found that the incidence of cardiovascular events was greater in patients with diabetes (16.4%) than in those with prediabetes (9.2%) or normoglycemia (8.5%) [71]. This indicates that patients with DM are at an elevated risk of atherosclerotic cardiovascular disease (ASCVD) and the risk is partly because of mixed dyslipidemia, i.e., increased plasma TGs, LDL-C, non-high-density lipoprotein cholesterol (non-HDL-C), and decreased HDL-C [72, 73]. In addition, alirocumab treatment targeting LDL-C 0.65–1.30 mmol/L caused nearly twice the absolute reduction in cardiovascular events in patients with diabetes (2.3%) compared with those with prediabetes (1.2%) or normoglycemia (1.2%) [71]. Another study found that treatment with alirocumab can significantly reduce the level of non-HDL-C, LDL-C, and apoB among patients with DM [74]. Thus, PCSK9 mAbs may effectively decrease the cardiovascular risk of patients with DM by reducing the level of circulating atherogenic particles including non-HDL-C, LDL-C, and apoB.

### Assessment of diabetogenic effects of PCSK9 mAbs

Yang et al. [75] found that liraglutide, a glucagon-like peptide-1 receptor agonist that is a widely used drug for DM, can suppress both blood glucose and PCSK9 expression. In a recent study on 539 stable CAD patients, individuals with lower plasma levels of PCSK9 had a higher prevalence of DM [76]. These findings indicate the possible role of PCSK9 in glucose metabolism. However, the clinically relevant effect of PCSK9 mAbs on glycemic parameters has not yet been observed. In patients with DM, alirocumab did not result in the change of glycemic parameters, i.e., HbA1c, fasting plasma glucose [71, 77, 78]. For instance, Colhoun et al. [77] found that in patients with DM, the change of HbA1c and fasting plasma glucose from the baseline at 24 weeks in the alirocumab group was similar to that in control group. Similarly, there was no significant difference in the change of glycemic parameters between the evolocumab group and the control group during a 52-week period [79]. In addition, plasma PCSK9 levels and treatment with PCSK9 mAbs are not associated with the risk of new-onset diabetes [71, 78, 80].

However, restricted by the limited number of participants and the relatively short follow-up period, these clinical trials rule out the long-term effects of PCSK9 mAbs on glycemic parameters and the risk of new-onset diabetes. There may be subtle diabetogenic effects of PCSK9 mAbs, but these potential effects can be easily managed by lifestyle changes and prescription of antiglycemic drugs. Moreover, PCSK9 mAbs provide substantial benefits for those at very high risk of cardiovascular events. Thus, any subtle or even moderate diabetogenic effect of PCSK9 mAbs, if ever established, is unlikely to outweigh the therapeutic benefit [81].

## Conclusion

The role of PCSK9 in atherosclerosis extends far beyond LDLR degradation. However, more research should be conducted on the structure of PCSK9 and the sites that PCSK9 targets to explore the molecular mechanism by which PCSK9 participates in non-classical pathways. Elucidating in detail the mechanisms by which PCSK9 affects atherosclerosis is important for guiding clinical medication of PCSK9 inhibitors. The addition of PCSK9 inhibitors induces a sharp decrease of LDL-C; hence, it is also important to detect whether other lipid metabolism processes have been affected. Moreover, whether the CRP level and platelets/fibrinogen level can be applied as indicative of yet another atherosclerotic mechanism of PCSK9 needs more exploration. Future studies on these topics may provide a theoretical basis for the development of novel drugs targeting PCSK9.

## Data Availability

Not applicable. No new datasets were generated for this review article.

## Abbreviations

LDL-C: Low-density lipoprotein-cholesterol
PCSK9: Proprotein convertase subtilisin/kexin type 9
mAbs: Monoclonal antibodies
siRNA: Small interfering RNA
EGF-A: Epidermal growth factor-like repeat A
LDLR: LDL receptor
SREBPs: Sterol regulatory element-binding proteins
PCSK9^−/−^: PCSK9 knockout
WT: Wild-type
TG: Triglyceride
TRL: Triglyceride-rich lipoprotein
SMCs: Smooth muscle cells
ARH: Autosomal recessive hypercholesterolemia
VLDLR: Very low-density lipoprotein receptor
LDLR^−/−^PCSK9^−/−^: LDLR and PCSK9 knockout
apoB: Apolipoprotein B
Lp(a): Lipoprotein(a)
RCT: Reverse cholesterol transport
ABCA1: ATP-binding cassette transporter A1
HUVECs: Human umbilical vein endothelial cells
ox-LDL: Oxidized-LDL
CAD: Coronary artery disease
MCP-1: Monocyte chemo-attractant protein 1
SRs: Scavenger receptors
CCR2: C–C chemokine receptor 2
VSMCs: Vascular smooth muscle cells
hs-CRP: High-sensitivity C-reactive protein
mtROS: Mitochondrial-derived reactive oxygen species
DM: Diabetes mellitus
ASCVD: Atherosclerotic cardiovascular disease
non-HDL-C: Non-high-density lipoprotein cholesterol
